# Urban Green Space: Creating a Triple Win for Environmental Sustainability, Health, and Health Equity through Behavior Change

**DOI:** 10.3390/ijerph16224403

**Published:** 2019-11-11

**Authors:** Hanneke Kruize, Nina van der Vliet, Brigit Staatsen, Ruth Bell, Aline Chiabai, Gabriel Muiños, Sahran Higgins, Sonia Quiroga, Pablo Martinez-Juarez, Monica Aberg Yngwe, Fotis Tsichlas, Pania Karnaki, Maria Luísa Lima, Silvestre García de Jalón, Matluba Khan, George Morris, Ingrid Stegeman

**Affiliations:** 1 Centre for Sustainability, Environment and Health, RIVM, 3720 BA Bilthoven, The Netherlands; nina.van.der.vliet@rivm.nl (N.v.d.V.); brigit.staatsen@rivm.nl (B.S.); 2 Institute of Health Equity, UCL, London WC1E 7HB, UK; r.bell@ucl.ac.uk (R.B.); matluba.khan@ucl.ac.uk (M.K.); 3 Basque Centre for Climate Change (BC3), 48940 Leioa, Spain; aline.chiabai@bc3research.org (A.C.); silvestre.garciadejalon@bc3research.org (S.G.d.J.); 4 Instituto Universitário de Lisboa (ISCTE-IUL), CIS-IUL, 1649-026 Lisboa, Portugal; gmtos@iscte.pt (G.M.); luisa.lima@iscte.pt (M.L.L.); 5 European Centre for Environment and Human Health, University of Exeter Medical School, Truro TR1 3HD, UK; S.Higgins@exeter.ac.uk (S.H.); geomorris55@hotmail.co.uk (G.M.); 6 Department of Economics, Universidad de Alcalá, Plaza de la Victoria, 2, E-28802 Alcalá de Henares, Spain; sonia.quiroga@uah.es; 7 Health Economics Group, University of Exeter Medical School, Exeter EX1 2LU, UK; p.martinez-juarez@exeter.ac.uk; 8 Department of Public Health Sciences, Stockholm University, Institutionen för folkhälsovetenskap, 106 91 Stockholm, Sweden; mayngwe@icloud.com; 9 Institute of Preventive Medicine Environmental & Occupational Health, Prolepsis, 7, Fragoklisias street, 151 25 Marousi, Greece; fotis.tsichlas@gmail.com (F.T.); p.karnaki@prolepsis.gr (P.K.); 10 EuroHealthNet, 1000 Brussels, Belgium; i.stegeman@eurohealthnet.eu

**Keywords:** green space, health, environmental sustainability, health equity, behavior change

## Abstract

Urbanization, costs of green space maintenance, and diminishing connection between people and nature all exert pressures on urban green space. This is regrettable as green space has the potential to create wins for environmental sustainability, health, and health equity. This paper explores this potential triple win and investigates how to increase the use of urban green space through behavior change. A narrative literature review was conducted and was supplemented with literature suggested by experts. Results show that creating well-designed green spaces and stimulating people to use them can indeed deliver this triple win. Providing accessible, attractive, well-maintained green space with room for socialization, and where people feel safe, may increase the opportunity and motivation of people to use it more often. Informing and educating people and organizing activities may increase capability (and motivation) to use green space. Since the use of green space depends on life stage, lifestyle factors and individual values, it is important to involve potential users in its design. We recommend a specific focus on those groups who may benefit most from the use of green space. More evaluation is needed to inform effective green space interventions and to assess related economic, social, and environmental benefits.

## 1. Introduction

Patterns of urbanization in many European cities put pressure on available urban green space, such as public parks, urban gardens, woodlands, children’s play areas, roadside verges, riverside footpaths, and beaches [1]. Densification of cities often results in the removal or degradation of existing green space in ways that are difficult to reverse. Over time, not only has the quantity of urban green space declined, but also the quality [2]. The pressure on urban green space is further increased due to the economic value of land—a landowner profits more from urban land developed for commercial and industrial and residential purposes than from land used for green infrastructures. We assume that the first will provide higher market benefits, compared to green spaces, although there are environmental disbenefits and damages. Green spaces will have lower market benefits, but will provide in turn aesthetic, environmental, leisure, social, and health benefits, for which people may be willing to pay [3,4,5,6]. Furthermore, the maintenance of urban green space costs money and available maintenance funds can be limited in times of economic crisis [7]. Lastly, humans are generally less connected with nature than in the past, which may result in a lower perceived value of green space. All these drivers threaten provision and use of green space in cities.

Fortunately, there are also positive trends that may limit this negative tendency. Most people still regard the presence of green space near their homes as very important [8]. More recently, green space has been recognized as supportive of healthy living in urban areas in Europe. For example, the current EU Biodiversity Strategy (Target II) contains a commitment, not only to better protect ecosystems but also to ensure greater use of urban green infrastructure [9]. The positive influence of green space on the quality of urban life, and the urban economy, is now widely recognized. Urban green space can improve urban vitality and, in doing so, offer quality of life benefits [10]. There is also a growing interest in so-called “nature-based solutions” (NBS), which presents nature as potentially beneficial for a wide range of societal challenges. In particular, NBS may help societies to simultaneously address a variety of environmental, social, and economic challenges in sustainable ways, with consequent benefits to the well-being of urban populations, as specified below [11].

In addition to the above, the degradation of urban green space can contribute to the burden of disease by exacerbating the effects of other adverse factors in the urban environment. These include air pollution, noise pollution, and heatwaves. Moreover, loss and degradation of green space diminishes an important restorative resource for the individual, and reduces opportunities to meet people and engage in physical activity in an attractive and relaxing environment [1]. It may also aggravate inequalities in a broader sense. Generally, house prices are higher where there is more green space in the immediate area [7,9,12]. Increasing house prices in greener neighborhoods may “push out” lower income groups.

We argue, here, that an urban green space has significant potential to contribute simultaneously to environmental sustainability, health and health equity—a triple win. This aligns with the notion of “ecological public health” [13] which recognizes the interconnections and interdependencies among human health, ecosystem health, and social equity [14]. Human health acquires a distinct significance in the context of global change. At the societal level, there is now a requirement to embrace a holistic vision including the physical, mental and social dimensions of well-being, as well as the links to the natural environment, equity and fairness. This aspiration emphasizes the importance of interactions between the individuals and their environment. In this sense, all the benefits produced by the natural environment provide the basis for a healthier population.

Although much has been published on positive aspects of green space (e.g., [15]), most publications describe the impact of green space, either on health, on environmental sustainability or on health inequalities. In this paper, we take a new approach that considers a combination of all three aspects. Furthermore, we argue that the central contribution of human behavior in creating this triple win has hitherto been neglected.

This paper aims to address this gap by exploring the role of individual behavior in stimulating the use of urban green space in order to create a triple win for environmental sustainability, health, and health equity. When using the term environmental sustainability, in this paper, we refer to the environmental impacts, in the places we live our lives today (the “here and now”), but also elsewhere and for generations yet to be born (the “there and then”). When referring to health equity, we mean the absence of avoidable differences in health between subpopulations. This has multiple origins, including the social determinants of health (e.g., income, education, working conditions, health care access, social inclusion).

This paper is based on work conducted in the Horizon2020 project INHERIT (INter-sectoral Health and Environment Research for InnovaTion) which places an emphasis on behavioral change policies in pursuit of the triple win. That project aims to identify ways of living, moving, and consuming that protect the environment, and promote health and health equity. This paper reviews and synthesizes past research assessing the potential of urban green space to create wins for environmental sustainability, health, and health equity. Central questions include:

- What can be done to stimulate change in an individual’s behavior to increase their use of urban green space?
- Can this increased use of urban green space lead to a triple win? In essence:
  What is the impact of urban green space on environmental conditions?
  What is the impact of urban green space on health?
  Are there differences in the health impacts of urban green space between subpopulations?

## 2. Materials and Methods

A narrative literature review was conducted in order to provide a broad understanding of how to increase the use of urban green space through behavior change and to assess the potential triple win. The international scientific literature published in English between 2006 and 2016 from the databases Medline, Embase, Scopus, and PsychInfo was analyzed. A multidisciplinary team from within the INHERIT project selected the relevant papers that emerged from the literature search and extracted information from these using a predefined template. This template required background information on the publication (reference and general abstract), and a summary of the interventions, of the determinants of green space use, and (where applicable) of the reported effects of green space. In the period immediately prior to submission of this paper, the search was complemented with more recent papers identified by INHERIT partners and, subsequently papers highlighted as important and relevant by reviewers of this paper. The assembled information was then synthesized using the three dimensions of the COM-B system (see Section 3.1) to assess ways to change an individual’s behavior to stimulate people to use urban green space. In addition, the information on the impacts of urban green space on environmental conditions, on health and on differences in health impacts between subpopulations was also synthesized.

## 3. Results

### 3.1. Changing Capabilities, Creating Opportunities, and Influencing Motivation to Stimulate the Use of Urban Green Space

An early output of the INHERIT project has been a conceptual model, the INHERIT model [16]. The INHERIT model is formed by integrating the eDPSEEA model—an established conceptual model in the field of environmental and health [13]—with the Behavior Change Wheel (BCW), a model from the field of behavioral science [17]. For more details, readers are referred to van der Vliet et al. [16]—also included in this special issue. The BCW is based on 19 frameworks of behavior change, whose common features were brought together in the COM-B system (capabilities, opportunities, motivation, and behavior). According to this unified theory of behavior change described by Michie et al., changing capabilities, creating opportunities, and influencing motivation are the three ways to change individual behavior. Capability is about being psychologically and physically able to perform a behavior. Opportunity is about having a physical and/or social environment that make a behavior possible or prompt it, for example by making it affordable, (socially) acceptable, and accessible. Motivation entails all brain processes that motivate someone to perform a behavior (over other behaviors) at the relevant time. It includes reflective processes, such as those that result in a conscious decision to go jogging in a local park twice a week or having a positive attitude towards engaging in green space. It also includes automatic, unconscious processes, which involve more impulsive emotional responses and habits. These three elements interact with each other [17]. In the following section, we apply this theory of behavior change with respect to the use urban green space.

#### 3.1.1. Improving Capability

Not all people are equally capable of using green space—they may not be aware of the presence of green space in their community or may not be physically able to use it. In addition, there are differences in the use of green space associated with sociodemographic characteristics such as income, education, age, and gender, and with characteristics related to health status or cultural background. Based on a US study, there are indications that the majority of park users are white, have (or are) children, and engage in vigorous activity [18]. Furthermore, participants from high socioeconomic status areas were found to use the local park more frequently than those from low socioeconomic status areas [19]. By extension, actions to stimulate the use of green spaces should focus on the people who seldom if ever use them. When seeking to involve hard-to-reach groups, it is important to build trust and provide a sense of structure and continuity, e.g., through enthusiastic staff and key workers who remain involved throughout the entire duration of a program [12].

It is important that people are aware of the green space in their surroundings and that they appreciate the value of it for their own activities. To raise awareness, clearly marked routes, good information, and facilities on the routes are needed [12]. The effectiveness of information also depends on when the information is provided. Moments in people’s lives when contexts change are particularly effective in breaking old habits and establishing new ones, and through this, changing habitual behavior. Thus, a good moment to impart information might be when people move to a new home or have children [20]. When someone has recently moved to a different area, it may be especially useful to inform that person about green space availabilities in their new neighborhood. Equally, when people have children, it may be of use to inform them about the available children’s activities in green space [21].

Organizing activities in green space can impact on health in both direct and indirect ways. Activities may stimulate people to go to green space [22,23] with direct positive implications for their health [24]. For example, Bang et al. evaluated an urban forest walking program in which 50 office workers performed a walking exercise over 5 weeks. The researchers showed that this program had positive effects on physical activity levels, health-promoting behavior, and quality of life. However, there were no statistical differences in depression, waist size, body mass index, blood pressure, or bone density between the case and control groups [25]. Longer term, regularly organized activities are more effective than activities that take place only once. Thorough market research is also an important success factor for green space activities, as are good staff, a program of events with clearly defined dates and locations, the personality of group leaders and the use of advertising to local people. Nurturing good partnerships and providing opportunities to suggest improvements or highlight problems are also important [12]. These activities may also make people—including, often, hard-to-reach groups such as deprived urban communities—become more aware of their own behavior related to health and the environment in a broader sense, and motivate them to adopt healthier and more pro-environmental behavior in other domains. Furthermore, those participating in activities in green space may feel more connected with nature in the longer run. In addition, people may feel a greater sense of belonging, learn new skills, and have increased self-esteem after participating in such activities [12,26]. Moreover, training participants to continue to run activities as volunteers, or providing signposting to relevant opportunities may increase the sustainability of the effect of the activity [12].

A study investigating the association between frequency of participation in volunteer events related to green spaces found that those volunteering most frequently were most concerned over environmental issues, had the most developed sense of environmental identity and reported the most pro-environmental behaviors. Frequent volunteers also felt personally more attached to their local environment, believed that their efforts helped to solve environmental problems, and enjoyed being part of community efforts [27].

Nature education may stimulate people to use green space. This may also enhance pro-environmental behavior and sustainable attitudes in adults. Guided group walks, and school gardens are examples of how being in a green space can be “fun” and bring children in closer contact with nature. Other examples include food gardens in which people grow their own food or the involvement of communities in organizing park activities [28].

#### 3.1.2. Creating Opportunities

As mentioned above, creating opportunity requires the development of a physical and social environment that is accessible, safe, and affordable and includes room for socialization. Moreover, it must be socially acceptable to perform particular behaviors in that space [17,29]. Availability, size, the connectedness of space, ease of accessibility, distance, quality, attractiveness, and maintenance, are features of the physical environment contributing to increased social interaction [19]. The presence of walking paths, shade, water features, lawns, birdlife, lighting, sporting facilities, and other amenities including playgrounds is also important in promoting increased use [26,30]. Spaces also need to be suitable for the activities people want to undertake there. The involvement of potential users in the creation or restructuring of green space is important if these needs are to be met [29].

The creation, maintenance, and the use of green space are closely connected. If people reduce their use of the public green space, the incentive to provide new spaces and maintain existing spaces is correspondingly diminished. When combined with a consequent decline in maintenance and quality, public spaces are less likely to be used, amplifying a vicious spiral of decline. There is also a need to create safe areas, to ensure these are used regularly by the general public, and to secure sufficient “eyes on the street” to deter criminals and maintain safety [10].

An illustration of the positive effect of the renovation of parks on their use is described by Tester and Baker [31]. Two parks were renovated in San Francisco in resource-poor neighborhoods in an effort to increase access to quality playing fields for young people and families and to increase physical activity levels. Both parks/playing fields saw more than a 4-fold increase in the average number of visitors per observation among most age groups. There was a significant increase in sedentary, moderately active, and vigorously active visitors in both playing fields. The authors suggested that both the structural and programmatic changes led to increased park visits [31]. Another example concerns the refurbishment of a park in Australia, with the establishment of a fenced leash-free area for dogs; an all-abilities playground; a 365 m walking track; a barbecue area; landscaping; and fencing, to prevent motor vehicle access to the park. There was a demonstrable increase from pre- to post-improvement in the number of park users and the number of people observed walking and being vigorously active. At the control park, overall use decreased over the same period and there were no observed differences in walking or vigorous activity [32].

#### 3.1.3. Influencing Motivation

Actions that support capabilities and create opportunities can influence motivation to use urban green space. Intuitively, people will be motivated to use green space if it is nearby, perceived as safe, well-designed and well-maintained. More recently, it has been asserted that green space may “seduce” people to exercise more, by offering an attractive environment. This may be especially important for people who are difficult to motivate. In such cases, the target is the peripheral, automatic route to influencing people’s behavior, the belief is that, by encouraging people to engage in physical exercise, there is the chance that physical exercise will become habitual for them over time [17]. As mentioned before, such behavior change can best be achieved at moments in people’s lives when contexts change and during periods of transition [20].

The environmental characteristics that motivate people to participate in physical exercise in urban green space depend strongly on the type of activity: work (including study), active transport (walking, cycling), and leisure (recreation, sport). Within the work domain, the greenness of the setting is thought to be of little importance for the amount of physical activity, when active transport is not taken into account. Natural features may lead people to favor walking or cycling over other transport modes by making routes to destinations more attractive. However, distance to destination, availability of suitable infrastructure (e.g., sidewalks, bicycle paths), and safety are more important factors [28].

Being nature-oriented appears to be a stronger motivator for park use than having a park nearby. Park users with stronger nature orientation also appeared to: (i) spend more time in their garden, (ii) be willing to travel further to green space, and (iii) make longer visits than park visitors with weaker nature orientation [33]. Another motivator for the use of green space is childhood experiences. From that perspective, it is important to bring children in contact with nature. This may additionally enhance pro-environmental behaviors and potentially engender more sustainable attitudes and behaviors in adults [1].

Having children or having a dog, are other important motivators for using green space (e.g., [34]). For example, a study among about 4000 adults in four European cities showed that compared with non-dog owners, dog owners walked more and spent more time in green spaces, especially those living in or near greener areas [35].

Analyzing what motivates individuals towards engaging with green space is an important issue [36]. In order to better understand the benefits provided by green space, the classification of ecosystem services (ES) made in the Millennium Ecosystem Assessment framework is useful [37]. This work classifies goods and services provided by the ecosystem into four categories (provisioning, regulating, cultural, and supporting), identifying, therefore, four main motivations linked to different uses and benefits of the ecosystem for human well-being [38].

#### 3.1.4. Variation in the Use of Green Space

The use of urban green space can vary due to differences in the quality of the green space, the intended use, and its social context [26]. What makes green space attractive to people depends on several factors relating to themselves as individuals. These include their life stage, aspects of lifestyle and their individual values. For example, parents of young children want safe and pleasant spaces for their children to play. People without dependent children want spaces for socializing with others and enjoyment of nature, while teenagers want places to “hang out” safely without being moved on by the police or other adults [39]. Therefore, it is important to consult or involve potential users in the design of green space. A review of nature-based and other interventions showed that interventions that adopt a participatory approach, i.e., where participants are actively involved in the design of the intervention (e.g., contributing to the design of a new park), appear to be more effective in changing behavior into a healthier and more pro-environmental one, than those that do not [12]. Involving people in the design and maintenance of green space may not only contribute to higher quality green space, but can also have positive effects on attitudes towards the (local) environment and on social cohesion [28].

The use of public (green) spaces varies according to the time of the day, day of the week, and season of the year, and is affected by what is offered in a particular place at a particular time. For example, children and young people tend to use public green spaces at the end of the school day, while young adults use them also at night [10].

#### 3.1.5. Combining Actions

Several studies indicate the importance of combining the actions outlined above to increase the use of urban green space. Thus, actions targeting quantity and quality of green space (“hardware”) may be combined with actions that promote awareness of availability, location, accessibility of local green space (“software”). Attracting under-represented groups requires more than simply physical changes to the environment. Supported activities are crucial [31]. Projects that combine technical/infrastructural approaches with education, training, and community-based interventions, are more likely to have a more profound and lasting effect on behavior [12]. For example, Hunter et al. found that interventions where physical activity programs are combined with a physical change to the built environment (e.g., restructuring green space) are likely to have the largest effect on physical activity [40]. In addition, as mentioned above, it is important that people feel safe, and that there is a sense of social cohesion and perceived integration. For example, the presence of unsupervised older children and adolescents may be linked with antisocial behavior and, for some, this may be a reason not to use green space. Accordingly, creating social cohesion in a neighborhood can impact positively on the use of green space [39]. At the same time an urban green space may also promote social cohesion where people feel connected to that place, and to the people who visit it. In addition, nature-oriented people may also experience heightened attention and mindfulness, which is linked to attention restoration [26].

Generally speaking, interventions to change behavior which are embedded within local community structures and social networks are more effective than national level approaches [12]. Yet, at the same time, local level initiatives can be facilitated by national level strategies that encourage and incentivize urban green space [28].

### 3.2. A Triple Win of Urban Green Space?

If people use urban green space, can this lead to a triple win of improved environmental conditions, better health, and greater health equity? In this section, we attempt to answer this question based on our review of the international scientific literature. 

#### 3.2.1. Environmental Impacts of Urban Green Space

If well-designed, urban green space—such as street trees, parks, green roofs, and facades—can help achieve reductions in temperature and air pollution in urban areas while simultaneously delivering diverse additional benefits such as biodiverse habitats and enhanced living and recreation areas [1]. Urban heat islands can increase urban temperatures by up to 12 °C compared to non-urban areas. This can exacerbate heat stress in city dwellers [9]. Trees can provide shade and reduce the demand for air conditioning during warm periods [1], thus reducing energy demand and promoting sustainability. A meta-analysis of the literature on the effect of urban parks on air temperature showed an average cooling effect of approximately 1 °C [1]. This effect exists up to 1 km from the park boundary with factors such as canopy density and the nature of air flows determining whether this cooling effect is achieved in practice [41]. The inclusion of water bodies in the green areas may enhance cooling effects [1]. Furthermore, green space may help to reduce the risk of flooding in periods of heavy rainfall by increasing water retention and infiltration, and reducing runoff [42]. Although green space is often presented as a solution to problems caused by climate change, it is also affected by it. Stressors—such as changed hydrology, low soil quantity and quality, fires and wind events, each detrimental to green space—may be aggravated by more frequent and intense weather events. Also relevant here, is the likelihood that some urban tree species will not adapt well to a changing climate and the vulnerability of unhealthy urban trees to insects and diseases [43].

Creating more green space in cities may increase the sequestration of CO_2_ to an, albeit limited, extent. The literature revealed that tree photosynthesis in urban green space is able to offset a fraction of the CO_2_ emitted from internal combustion engines [44] potentially helping to mitigate the effects of climate change. Realistically, however, the extent of any possible mitigation may be relatively inconsequential given that levels of urban green space is generally quite limited [28]. Green infrastructure holds potential to promote the reduction of CO_2_ emissions indirectly by changing behavior, for example by facilitating beneficial mobility choices such as walking and cycling. This has additional potential to reduce the other traffic-related air pollutants in the urban environment. Through another mechanism, urban green spaces may act as buffers and provide valuable oases where air quality is higher than surrounding areas. Although a topic of debate, some commentators state that trees and other vegetation may reduce levels of some pollutants, including gases and particulate matter. Given their large surface resistance, trees have the potential to reduce air pollution by dry deposition, leading to improvements in human health and well-being, although this impact seems to be limited [45]. Paradoxically however, trees may also contribute to air pollution by releasing hydrocarbons and reducing the opportunity for dispersal of certain pollutants such as low-level ozone. Careful selection of species [46], design of planting configurations with regard to airflows, shade, other impacts, and maintenance of urban vegetation all need to be optimized to generate the benefits to air quality [15]. Furthermore, the cooling effect of vegetation, through shade and evapotranspiration, can help generate airflows, and disperse the concentration of pollutants [9].

Well-designed urban green space can buffer noise, or at least the negative perception of noise emanating from non-natural sources, such as traffic, thus providing relief from city noise [1]. Vegetation has been considered as a means to reduce outdoor noise levels, mainly in areas with high volumes of traffic. It can impede noise propagation by absorbing or diffracting noise [9]. A different effect of green and blue space on noise perception is the effect of “natural” noises in masking noise from for example traffic (e.g., sounds of water fountains or birds) [1].

#### 3.2.2. Health Impacts

Various reviews showed strong evidence of significant positive associations between green space and both physical and mental health [1,47]. Health impacts include perceived general health, perceived mental health and all-cause mortality, cardiovascular disease mortality, reduced cardiovascular impacts, reduced prevalence of type 2 diabetes, and reduced adverse pregnancy outcomes [1]. While many studies on green space and health use the amount of green space as the key indicator, there are increasing indications that the accessibility, type, quality, and context of green space should also be considered in the assessment of relationships between green space and human health and well-being (e.g., [48]). People’s perception is another important indicator of green space quality [8,49]. Green spaces have different meanings for people, both positive—related to identity, community, restoration, safety, and freedom/unity; and negative—related to maintenance and crime and conflicts associated with inequality and access [50]. The association between urban green space and health seems to vary across a whole range of dimensions the (type of contacts, environments, life course, age, gender, social groups, level of physical activity) (e.g., [7,11,15,25,26,27,29,44,45,46,47,48,49,50,51,52,53,54]). A recent study by Bratman et al. pointed out that mental health benefits may vary by socio-demographic variables (socio-economic status, gender, age, occupation), preferences, personality traits, culture, and residential location. In addition, the type of interaction with nature, and the form of sensory input (e.g., visual, olfactory, auditory, or tactile) may have different impacts on mental health [47].

Multiple pathways have been offered in explanation for the impacts of green spaces on health but many are still imperfectly understood [15,55]. Hartig et al. presented, in their review of reviews, a comprehensive framework, suggesting that (contact with) nature can have an influence on stress levels, social contacts, physical exercise, and air quality, and, in that way, may impact on health and well-being [15]. Others suggested additional pathways, including ones related to adverse impacts of green space (e.g., [1]). We discuss the main suggested pathways below.

##### Stress and Subjective Well-Being

Many studies report a positive association between nature experience and stress reduction and improved subjective well-being. This includes positive effects such as happiness; a sense of meaning and purpose in life; improved manageability of life tasks; and a decrease in mental distress. Also evidenced are improvements in cognitive function; memory and attention; impulse inhibition; children’s school performance; and imagination and creativity [47].

Green space can reduce stress and increase subjective well-being in two general ways. First, natural areas and features can reduce exposure to challenging environmental conditions by increasing distance to stressors and/or decreasing their perceptual salience. For example, green spaces between dwellings and heavily trafficked roads can reduce noise annoyance for residents, vegetation can conceal displeasing structures, and landscaping around housing can maintain privacy and avoid a sense of crowding. Second, nature can help people restore their adaptive resources to cope with stress. The extent to which people are restored by urban green spaces depends on individual perceptions and needs as well as physical characteristics of the setting [56]. In addition, green spaces can create sense of belonging and decrease social isolation, which may have a stress-buffering effect [26]. Escape from physical and social stressors has long been described as an important motive for recreation in natural areas. Appreciation of nature—for its beauty, symbolic qualities, and other valued attributes—is another important motive [15].

The greater part of the evidence for this pathway relates to the short-term restorative benefits of single encounters with, or experiences in, nature and are based on experiments. For example, Song et al. showed in their studies of two groups of men—numbering 17 and 23, respectively—that heart rate was significantly lower while walking in an urban park compared to walking along a city street. Furthermore, the urban park walk led to higher parasympathetic nervous activity and lower sympathetic nervous activity compared with the walk through the city street. The men exhibited significantly lower levels of negative emotions and anxiety after the walk through nature and felt more relaxed, comfortable, and natural [57,58,59]. Li et al. studied 16 healthy adult male subjects who took day trips to a forest park in the suburbs and to an urban area of Tokyo for 2 h in the morning and afternoon on a Sunday. They also found that habitual walking in forest environments may lower blood pressure by reducing sympathetic nerve activity and increase parasympathetic nerve activity [60]. Gidlow et al. compared psychological and physiological responses of 38 unstressed individuals to self-paced 30 min walks in three environments: residential (urban), natural (green), and natural with water (blue). Mood, cognitive function, restoration experiences, salivary cortisol, and heart rate variability (HRV) were measured at baseline (T1) to T2 (end of the 30 min walk), and T3 (30 min after leaving the environment). Stress reduction in all environments pointed to the salutogenic effect of walking, but natural environments conferred additional cognitive benefits lasting at least 30-min after leaving the environment [61]. 

A study by Berman et al. in 20 people with major depressive disorder (MDD) pointed out that a working-memory capacity and positive affect emerged significantly after the nature walk relative to the urban walk [62]. Another study by Triguero-Mas, in which 26 people with indications of psychological distress were exposed, in groups, to green, blue, and urban environments in Catalonia for a period of 30–180 min, found lower total mood disturbance (TMD) and salivary cortisol amongst those exposed to the green environment compared to those exposed to urban environments. These findings achieved statistical significance. There were also statistically significant favorable changes in heart rate variability indicators in the environment with a blue space. Notable also, was a finding that physical activity and self-perceived restoration experience partially mediated the associations between exposures to green and blue spaces and TMD. In addition, physical activity and air pollution partially mediated the associations between exposures to green and blue spaces and heart rate variability [63].

In addition to these experimental studies, several epidemiological studies showed that nature acted positively to reduce stress. For example, a study of Stigsdotter et al. among 21,832 Danish adults showed that respondents living more than 1 km away from green space had 1.42 higher odds of experiencing stress than respondents living less than 300 m from green space. Those living more than 1 km from green space also reported poorer health and health-related quality of life than respondents living closer to green space [59]. Another study by van den Berg et al. among 4529 Dutch respondents to the second Dutch National Survey of General Practice (DNSGP-2) revealed that respondents with a high amount of green space within a 3 km radius were less affected by experiencing a stressful life event than respondents with a low amount of green space in this radius [64].

##### Physical Activity

The outdoor environment may offer suitable spaces for physical activity. Several studies in various countries have demonstrated that access to, and use of, green space stimulates recreational walking, increases physical activity and reduces sedentary time. For example, Lachowycz and Jones found in their study on green space and self-reported levels of walking among 165,424 adults across England, that there were between 13–18% more days of recreational walking in the greenest quintile compared to the least green quintile [55].

However, the evidence for the association between green space and physical activity is mixed, and this heterogeneity has been well summarized by recent systematic reviews [13,65]. Reasons may include the fact that large amounts of green space tend to go together with (a) greater distances to destinations, (b) higher levels of car ownership, and (c) better availability and lower cost of car parking spaces in the vicinity of the home.

The association between green space and physical activity is complex. For example, Mytton et al. found a positive association between green space and physical activity levels in general. However, when analyzing green space and types of physical activity normally associated with green space (gardening and do-it-yourself, and occupational physical activity), no association was found [66]. On the contrary, other studies, such as Picavet et al., found an association only when differentiating by type of activity and by type of green space, it appeared that more urban green was associated with more time spent on bicycling and sports, and less time spent on gardening and odd jobs. For agricultural green space, it was the other way around [67].

The effects of urban green space on physical activity and on restoration and mental health are often studied in combination. As described above, walking or running in green spaces is associated with a more restorative experience when compared to the same exercise in a city street [1]. Barton and Pretty’s analysis of 10 United Kingdom studies showed multiple mental health benefits from physical activity in green environments [68]. Mitchell’s study of the Scottish population showed an association between physical activity in natural environments and a reduced risk of poor mental health, while activity in other types of environment was not linked to the same health benefit [1].

White et al. estimated the total annual amount of adult recreational physical activity in England’s natural environments and assessed its implications for population health. These calculations revealed that approximately 8.23 million adults (19.5% of the population) made at least one “active visit” (i.e., ≥30 min, ≥3 metabolic equivalents (METs)) to natural environments in the previous week, resulting in 1.23 billion “active visits” annually. In addition, an estimated 3.20 million of these adults reported meeting recommended physical activity guidelines (i.e., ≥5 × 30 min a week) fully, or in part, through such visits. Active visits by this group were associated with an estimated 109 Quality Adjusted Life Years (QALYs) annually. Assuming the social value of a QALY to be £20,000, the annual value of these visits was approximately £2.18 billion. Results for walking were replicated using the WHO’s Health Economic Assessment Tool [69].

Health benefits of increased physical activity are largest among those who initially had the lowest levels of physical activity [15].

##### Social Cohesion

Social cohesion refers to the interpersonal dynamics and sense of connection among people.

It can improve physical and psychological health. The presence of urban green spaces can stimulate positive social interactions that contribute to social cohesion, which in turn may also increase physical activity and may have a stress-buffering effect. The potential positive social experiences may increase social capital, sense of community, and may empower people [26]. However, the relatively few available empirical studies on the topic show mixed results. Sugiyama et al., for example, found perceived social coherence and local social interaction to be associated with the perceived greenness of the neighborhood [70]. De Vries et al. [71] found an association between streetscape greenery and perceived social cohesion at the neighborhood scale, both for the quantity and, even more strongly, for the quality of greenery. Community gardens may also increase social ties in a neighborhood [15]. A study by Maas et al. using data from 10,089 Dutch residents found that less green space in people’s living environment coincided with feelings of loneliness and with a perceived shortage of social support [72]. Ruijsbroek et al. found in their study in four European cities that more cohesive neighborhoods were greener and had better quality green space in two of the four cities. Stronger neighborhood attachment was found in neighborhoods with better (perceived) quality green space in three of the cities. No association was found with social contacts [73].

Social well-being may not be beneficially affected by green and open space that is perceived as unsafe or where people engage in antisocial behavior, although proper management and maintenance can address these problems. There is also some evidence that the provision of new green spaces in disadvantaged neighborhoods (e.g., greening of vacant lots) can reduce crime [1].

##### Immune Functioning

Kuo suggested a central role for enhanced immune functioning as a pathway between nature and health [1]. There are several potential explanations for this pathway. Firstly, Japanese studies have demonstrated associations between visiting forests and beneficial immune responses, including expression of anti-cancer proteins [1]. This suggests that immune systems may benefit from relaxation provided by the natural environment, but also through contact with certain physical or chemical factors in the green space. In addition, it has been shown that children with the highest exposure to specific allergens and bacteria during their first year were less likely to have recurrent wheezing and allergic sensitization [1]. A further suggested pathway is that social support can buffer changes in neuroendocrine, cardiovascular, and immune function [26]. Assuming that social support can be improved by an increase in positive social interactions in green space, visiting green space may also have a beneficial effect through this pathway. Another suggested immunological pathway is through exposure to diverse microorganisms in green space which can play an immunoregulatory role [1]. For example, Hanski et al. found in their study among 118 adolescents in eastern Finland that, compared with healthy individuals, atopic individuals had lower environmental biodiversity in the surroundings of their homes and significantly lower generic diversity of gammaproteobacteria on their skin, that help to prevent people from having allergic reactions [74]. These results raise fundamental questions about the consequences of biodiversity loss for both allergic conditions and population health in general.

##### Increased Exposure to Sunlight

The WHO [1] discussed the health effects of increased exposure to sunlight by spending time in green space. This may have both positive effects (vitamin D from sunlight, improved sleep) as well as negative effects (exposure to dangerous levels of ultraviolet (UV) light, causing skin cancer). Exposure to sunlight is especially important for northern Europeans whose environment lacks high levels of sunlight for significant parts of the year, and for older people, since the ability to synthesize vitamin D decreases with age. De Rui et al. found that vitamin D levels were significantly higher in older people who engaged in outdoor activities, than in those who did not. The levels were particularly high for those who cycled or gardened (De Rui et al. in: [1]).

##### Adverse Impacts

Urban green space may also have adverse impacts on health. Health risks from green space include vector-borne diseases, which are transmitted by arthropods, such as ticks (e.g., tick-borne encephalitis and Lyme disease), mosquitoes (e.g., Chikungunya fever and Dengue fever), or sand flies (e.g., visceral leishmaniasis). Lyme disease, in particular, has increased in Europe in the 21st century, and this has been associated with urban green space and increased populations of animal hosts, such as deer, as well as with climate change and milder winters in northern Europe. Furthermore, contamination of urban green space with dog or cat feces poses another risk. Ingestion of dog feces by young children can lead to toxocariasis, with serious illness and blindness possible in rare circumstances. Cat feces may cause severe neurological damage in children born to mothers who were infected for the first time during pregnancy [1].

Many trees and plants release pollen, which can aggravate allergies. An increasing proportion of the urban population is susceptible and allergic to tree-derived pollen. Therefore, identifying tree species that are most responsible for allergic reactions is important [44].

Living close to green space may be associated with elevated exposure to pesticides and herbicides especially if they are used in inappropriate ways and at excessive levels. The insecticides malathion and diazinon and the herbicide glyphosate, which are used to control weeds in urban parks, may be carcinogenic in humans [1]. Another risk of using urban green space, is the risk of accidents and injuries, resulting from tree or tree or branch fall or trips slips and falls by individuals and drowning, by people engaged in physical activity [1].

#### 3.2.3. Health Inequalities—Impacts of Urban Green Space on Different Subpopulations

Several studies report differential health effects of green spaces among different subpopulations, related to socioeconomic status, and health status (e.g., [1,47]).

##### Effects in Deprived Communities

European data indicates that socially disadvantaged people often live in places with less access to public green space [75,76]. This was confirmed by Schüle et al., based on their systematic review of quantitative observational studies conducted in the 53 Member States belonging to the WHO European Region and published in peer-reviewed journals. Ecological studies consistently show that deprived areas have lower green space availability than more affluent areas. However, associations in cross-sectional studies on the individual level were mixed and dependent on the type of socio-economic indicator and the green space measures applied [77]. Despite having greater access to public green space, those with higher educational attainment complained more often about lack of access to recreational or green areas than those with lower levels of education [75].

Mitchell and Popham demonstrated that health inequalities related to income deprivation in all-cause mortality and mortality from circulatory diseases were lower in populations living in the most green areas [54]. Lachowycz and Jones found that the relationship between green space access and reduced mortality was only apparent in the most deprived areas [34]. A study by Ward-Thompson et al. in Scottish deprived areas showed that access to green space in these neighborhoods was a significant predictor for stress reduction. The authors mentioned a sense of place belonging, reduced sense of social isolation, and opportunities to manage or mitigate stress and maintain year-round healthy activity in green space, as elements to explain the positive effects [78]. Provision and maintenance of appropriate green space in urban areas may make an important contribution to reducing health inequalities and may buffer some of the effects of stressors such as unemployment [1,78].

##### Effects in Different Age Groups

A study by Lachowycz et al. showed that although children carried out the majority of their activity in non-green environments, urban green spaces—both public and private—are valuable resources for children’s play and physical activity [79]. Adequate exposure to green space for children may not only facilitate healthy development in childhood but may also provide long-term health benefits through adulthood. It may also stimulate the development of gross and fine motor skills as well as cognitive, emotional, social, and physical development in children [1]. In this way, it may lead to better health and a better ability to maintain healthy lifestyles in adulthood. Contact with nature may improve attentional function in children with attention deficit disorder and enhance self-discipline in children without a diagnosis (e.g., [80]). Some research suggests that restorative childhood contact with nature can provide cumulative benefits with far-reaching developmental significance [15]. Dadvand et al. found that living in greener residential areas and proximity to forests was associated with less sedentary time and reduced children’s risk of being overweight or obese, but for other green space measures, the effect was weak. For asthma, the authors found both positive and negative associations with green space indicators (Dadvand et al., in: [1]). A study of Flouri et al., using data from 6384 children participating in the Millennium Cohort Study, revealed that access to a garden and use of parks and playgrounds were related to fewer conduct problems (problems related with antisocial behavior), and fewer peer and hyperactivity problems. Furthermore, poor children aged 3–5 years old and living in urban neighborhoods with more greenery had fewer emotional problems than their counterparts in less green neighborhoods [81]. Markevych et al. also found that more access to urban green spaces was associated with fewer behavioral problems in a study population of 1932 10-year old children living in Munich, Germany [82]. By using green space, children also learn to deal with the physical risks of nature. Furthermore, public urban green space plays an important role in children’s and young people’s social networks, including friendships across cultures, thus promoting social inclusion [1].

Green space can also be beneficial to older people. For example, Sugiyama and Ward Thompson demonstrated an association between the quality of neighborhood open space and increased walking by older people (65 years and older) in the United Kingdom [1]. In a large Australian study among 260,061 Australians over 45 years old, those in the greenest neighborhoods were at a lower risk of psychological distress (odds ratio 0.83, 95% CI: 0.76, 0.92) and were less sedentary compared to residents of the least green areas (odds ratio 0.81, 95% CI 0.77, 0.87). Furthermore, the effect on mental health was strongest among those most physically active [52]. Older people derive considerable pleasure and enjoyment from viewing and being in nature which, in turn, has a positive impact on their well-being and quality of life [83]. Those living in inner-city neighborhoods also benefit from the presence and use of green spaces, which promotes social ties and a sense of community [1]. Social contact is known to be important for health and well-being, especially for older people, where social isolation has been significantly associated with increased mortality [1]. Bell et al. [84] pointed out that older age is associated with periods of significant change, particularly relating to retirement, personal and spousal health, caring duties, and bereavement. Green spaces can alleviate some of the negative impacts of such transitions on personal well-being. For example, studies have highlighted that older participants in group-based nature conservation and gardening activities appreciate the opportunities gained for structure and routine; meaningful social interaction and the development of stronger communities; a sense of achievement, pride and ownership; and the forging of new social identities (e.g., [85]). In addition, van den Berg et al. found that allotment gardeners of 62 years and older scored better on all measures of health and well-being than neighbors in the same age category without an allotment garden, living next to the home addresses of allotment gardeners [86].

Whear et al. showed that people with dementia living in care homes, but also their family, and staff, alike, appreciated the presence of a garden that both allowed for relaxation, and could stimulate activities and memories. The presence of a garden may be relaxing and calming, while also providing an opportunity to maintain life skills and habits. In addition, physical activity may have a role in slowing cognitive decline and in reducing falls. It also provides a normalizing context for interactions with staff and visitors [87].

##### Effects on Mental Illness

For people with mental illness living in urban areas, physical activity in green space may be particularly beneficial [1]. We described above, the positive effects found in studies by Berman et al. [62] and Triguera-Mas et al. [63] (p. 8–9, lines 386–397). In addition, Maas et al. demonstrated a strong protective effect on depression and anxiety of green space within 1–3 km of the home [72]. This was confirmed in a study in the United States, that found that higher levels of neighborhood greenery were linked to lower levels of depression, anxiety, and stress (Beyer et al., 2014 and Reklaitiene et al. 2014, in: [1]). They showed that, for individuals who regularly use parks, closer proximity of their home to the nearest park was associated with reduced odds of self-reported symptoms of depression. Based on a questionnaire survey among 3748 adults in four European cities, van den Berg et al. found that people who spend more time in green spaces have better mental health and vitality. This effect was modified by the level of education and childhood nature experience [88]. Based on a meta-analysis of 10 UK studies including 1252 participants in total, Barton and Pretty found improved self-esteem and mood among people exercising in green space with the presence of water generating still greater effects. Self-esteem improvements were greater in people with a mental illness [68]. Bratman et al. found that exposure to green space reduces neural activity in the subgenual prefrontal cortex and alleviates symptoms of depression [89].

## 4. Discussion

### 4.1. Summary and Lessons Learned

This paper analyzes the scientific literature to find answers to two main questions: What can be done to change an individual’s behavior to stimulate people to use urban green space? Can this increased use of urban green space lead to a triple win of improved environmental sustainability, improved health, and improved health equity? 

To answer the first question, we used the COM-B model, part of the Behavioral Change Wheel from Michie et al. According to this model, increasing opportunities, motivation and capabilities are important to change behavior [17]. We found that not all people are equally capable of using green space, either because they are not aware of the presence of green space or, for various reasons, are unable to. There are also differences in green space use related to personal characteristics. Empowering people and raising their awareness may promote capability. Clear signage, facilities on the route, and good information regarding available green space and the activities that take place there may all help to stimulate use. Also important in securing engagement is education about green space and engendering a sense of fun around its use. Organizing activities in green space, preferably with the involvement of the users, may not only increase their capability to use this area, it may also increase their sense of belonging and self-esteem, and can stimulate the acquisition of new skills. Having skilled and enthusiastic staff to lead these activities can help to build trust and continuity and assist when seeking to engage difficult to reach groups. These green space activities and nature education may also result in more sustainable, pro-environmental behavior.

Opportunities and motivation to use green space can be improved by providing a space that is accessible, well-maintained and has room for socialization. There is frequent reference to the safety of green space, emphasizing the need to work on social quality and social cohesion in neighborhoods in parallel with actions which shape and maintain the physical domain.

Availability, size, the connectedness of space, ease of accessibility, distance, quality, attractiveness and maintenance, are features of the physical environment that increase the opportunity and contribute to the actual use of green space. Furthermore, usage is likely to be increased by the presence of walking paths, shade, water features, lawns, birdlife, lighting, sporting facilities, and playgrounds. Green spaces exhibiting such features may motivate people living nearby to use them (“automatic motivation”). There is a key message around making green space suitable for the activities people want to undertake there. Since the activities and attractiveness of a place to an individual depend on their life stage, lifestyle, and individual values, it is important to take these into account and involve people in designing, shaping, and maintaining green spaces in their neighborhood. Such involvement and engagement can impact domains far beyond green space and result in a much broader neighborhood benefits. People may feel more responsible for the green space and, in a broader sense, for their living environment. Furthermore, there may be an enhanced sense of belonging and improved social cohesion.

Being nature oriented and having positive childhood experiences of nature motivate green space use. This emphasizes the importance of bringing children into contact with nature. It is also important to combine actions to develop and maintain green infrastructure with activities to raise awareness of green spaces—their availability, location, and accessibility—and the uses to which local green space is put. To secure the largest long-lasting effects, such measures should be embedded within local structures and social networks.

Regarding the second research question, the literature generally supports our hypothesis that the use of green spaces can lead to a triple win of improved environmental sustainability, improved health and improved health equity. Whether this happens in practice, depends on several conditions set out below, together with certain other issues we identify as knowledge gaps, which need to be addressed in future research:

1. Urban green space may improve environmental sustainability—if well-designed, attractive, accessible, and well-maintained—but only when implemented on a significant scale. Only then it may: (i) reduce temperatures in cities and, in so doing, counter the effect of urban heat islands and extreme rainfall; (ii) have a substantial effect on reducing atmospheric CO_2_; iii) reduce exposure to air pollution and noise through oases or buffers with clean air and attractive soundscapes. It may encourage more sustainable and healthy behavior, by making it attractive for people to, e.g., cycle or walk, but this comment relates mainly to recreational cycling and walking. The question is, to what extent is an aspiration to introduce and maintain quality urban green space on a significant scale realistic, against a backdrop of seemingly relentless urbanization and densification of cities, and the costs implicit in greenspace solutions? Furthermore, there are many assumptions around the beneficial effects of urban green space on the reduction of air pollution, CO_2_, noise, and heat stress, yet quantitative evidence of these effects and the effects of different designs remains quite limited.
2. There is increasing evidence that urban green space has a positive effect on mental and physical health and potentially improves the immune functioning of the body (e.g., [1]). At the same time, urban green space may also have potential to engender adverse health impacts, through elevated exposure to allergens (pollen) and risk of infectious diseases, skin cancer from excessive UV exposures [1]. However, with diligence, good practice, good design and maintenance, many of these adverse impacts may be prevented. In practice, balancing the positive influences of more and better green space against the potentially negative implications may prove difficult. We consider that available knowledge on benefits and risks is not always widespread, or widely available to professionals involved in, for example, urban design.
  The available studies on green space and health are in many respects heterogeneous. Such heterogeneity makes it particularly demanding to conduct the proper meta-analyses necessary to quantify all the health impacts of green space on health. Indeed, since the association between green space and health seems to differ so markedly between different locations, contexts, type of health impact, and method, it is questionable to what extent it is possible to establish a single generalized association for urban green space and the different health outcomes. Many factors affect the relationship and navigating in such complexity to deliver effective policies involves understanding and modeling these factors.
  Despite the many identified and hypothesized pathways linking greenspace and its characteristics to health and well-being, much remains to be uncovered about these and how they interact. The strongest evidence currently seems to point towards the pathway of stress reduction, restoration, subjective well-being, and mental health. For the other pathways, the evidence is less straightforward and more mixed. Moreover, some of the health effects described in relation to stress may be a result of interaction with physical activity. Physical activity may also reduce stress levels and, via that pathway, favorably affect both physical and mental health (e.g., [70]). In addition, there may be synergistic effects [15]. For example, people who are socially integrated may more often be stimulated to engage in physical activity and even more so when green and blue spaces are easily accessible.
3. Deprived communities, children, older people, people with mental health problems, and pregnant women seem to be the greatest beneficiaries of urban green space. Provision and maintenance of appropriate green space in urban areas may make an important contribution to reducing health inequalities and may buffer some of the effects of stressors such as unemployment. However, the literature shows that people from deprived communities often have less access to green space.
  Furthermore, when neighborhoods are made “greener”, housing prices often increase, which may “push” lower-income people out of these neighborhoods. Considering the health and other benefits, particularly for residents of deprived neighborhoods, it is recommended that policymakers “green” these neighborhoods but create the opportunity for more deprived groups to continue living there. This might be achieved by, for example, providing social housing in green spaces. Needless to say, these “greening” measures are only one among many measures needed to improve living conditions for disadvantaged people. They are not a substitute for other policy initiatives to improve these conditions.
  The targeting of other societal groups is also important. For example, to secure a good and healthy start in life, providing healthy environmental conditions for pregnant women is important, as is bringing children in contact with nature. This is not only because of the positive effects on, e.g., obesity, ADHD, concentration, allergic sensitization, and behavior, but also because active and healthy lifestyles provide benefits in later life. Furthermore, experience of nature in early life seems to play an important role in later use of green space and pro-environmental behavior. Older people are another important target group. Activities related to green space may keep them physically active, provide social contacts, help to structure their day-to-day lives, and improve their quality of life in general. For people with mental health problems, it may reduce symptoms like depression, anxiety, and stress, and increase self-esteem.
  Considering all these health benefits, green space has the potential to reduce health care costs, a factor which can also make a green space of interest to health insurance companies. Several health professionals already encourage the use of green space through reimbursing or subsidizing “therapeutic” activities in nature, and working with health professionals to prescribe such activities. Such initiatives are in their initial phase, and there is significant potential to expand and evaluate these.
  Just as the groups discussed are potentially the beneficiaries of green space, it is necessary to record that they are often also more vulnerable to risks associated with its use. Accordingly, it is important to educate and protect these groups about the risks. Involving them in the design, and maintenance of urban green space, and in the activities that take place, it seems likely to improve self-esteem and enhance their sense of belonging.

### 4.2. Directions for Future Research

Building on the issues raised above, there are several directions we recommend for future research.

Related to behavior change, this paper has placed special emphasis on the individual behavior of green space users. However, it is implicit that creating and maintaining urban green space requires several stakeholders to work together in the spirit of co-operation. These might include protected area authorities, green NGOs, city or regional authorities, various health sector stakeholders, social stakeholders, policymakers, and funders at all levels. The role and behavior of these stakeholders is of itself worthy of exploration to gain insight, for example into what motivates investment in green space, or measures which stimulate people to go there and, ultimately, to secure a transition to healthy and sustainable behavior of the population in a broader sense.

In relation to the triple win, we have already noted several knowledge gaps. More quantitative evidence is needed on the beneficial effects of urban green space on air pollution and CO_2_, on noise and on heat stress, in addition to evidence on the effects of different green space designs. This requires multidisciplinary research in which environmental (health) scientists, spatial designers, and green professionals work together contributing specific “pieces of the puzzle” that together may reveal new insights.

What elements related to green space can explain the beneficial health aspects? We have indications that, next to the presence of green space, matters such as quality, accessibility, safety, variety, facility provision, and acoustic quality are all important. In addition, how green space is used and how it is perceived is of equal importance. Identifying more accurately what indicators are of relevance, has potential to reveal the mechanisms underlying the relationship between green space and health. Much still needs to be discovered regarding the underlying pathways or mechanisms and, not least, how these interact. These pathway’s mechanisms and interactions are key targets for collaborative scientific enquiry.

From this study, we have learned that green space is potentially very valuable to deprived communities, children, older people, people with mental health problems, and pregnant women. Future research should investigate how we can improve capability, opportunities and motivation of these groups to maximize their use of green space. There is a particular need to engage hard-to-reach groups.

There are many examples of inspiring policies and interventions targeting green space and its potential users yet only a few of them have been evaluated. As a result, the effect of these policies and interventions and not least, their (economic) benefits are largely unknown. There is a clear need for longitudinal evaluations to identify prerequisites for success and to assess the long-term outcomes for behavior and the related environmental sustainability and health outcomes. Since combined actions seem to have the largest effect, it is of particular interest to evaluate instances where this occurs. In addition to local, small-scale intervention, there is a need to evaluate interventions (e.g., policy programs) at a more systemic level [12], since actions at that level are intuitively necessary to secure an optimal triple win.

There is also a need for cost–benefit analyses related to this topic. This is of particular interest to decision makers, and, by extension, important if green space is to become a core element in the policy agenda. Among the challenges mentioned above, are the lack of universal quantitative impacts of green space on health and the limited understanding of the importance of local context. Furthermore, studies need to identify the distinction between short-term and long-term effects and offer greater insights into who “pays” and who “receives” the benefits. 

## 5. Conclusions

Providing green space that is accessible, attractive, well maintained, with room for socialization, and where people feel safe may provide the opportunity and motivation for people to use green space more often. Informing and educating people about urban green space and organizing supportive activities in green space that benefit health may increase people’s capabilities to use it. Since the attractiveness of a place and of the activities offered depends on life stage, lifestyle factors, and individual values, it is important to take these into account and involve potential users in the design. Particular attention should be paid to groups who may benefit most from green space, such as deprived communities, children, older people, people with a mental illness, and pregnant women. A broad multidisciplinary collaboration and a combination of actions are needed to improve the use of green space and for this to have a long-lasting effect. Creating well-designed green spaces and encouraging people to use it can provide a triple win, by improving environmental sustainability, improving health, and improving health equity. Despite a large number of inspiring policies and practices, only a few have been evaluated. More evaluation, both qualitative and quantitative, is needed to provide further insight into what works.
